# Graphene Bilayer as a Template for Manufacturing Novel Encapsulated 2D Materials

**DOI:** 10.1021/acs.nanolett.4c03654

**Published:** 2024-10-04

**Authors:** Arkady V. Krasheninnikov, Yung-Chang Lin, Kazu Suenaga

**Affiliations:** † Institute of Ion Beam Physics and Materials Research, Helmholtz-Zentrum Dresden-Rossendorf 01328 Dresden, Germany; ‡ The Institute of Scientific and Industrial Research (ISIR-SANKEN), Osaka University, Osaka 567-0047, Japan; ¶ Nanomaterials Research Institute, National Institute of Advanced Industrial Science and Technology (AIST), Tsukuba 305-8565, Japan

**Keywords:** 2D materials, encapsulation, intercalation, high-resolution transmission electron microscopy

## Abstract

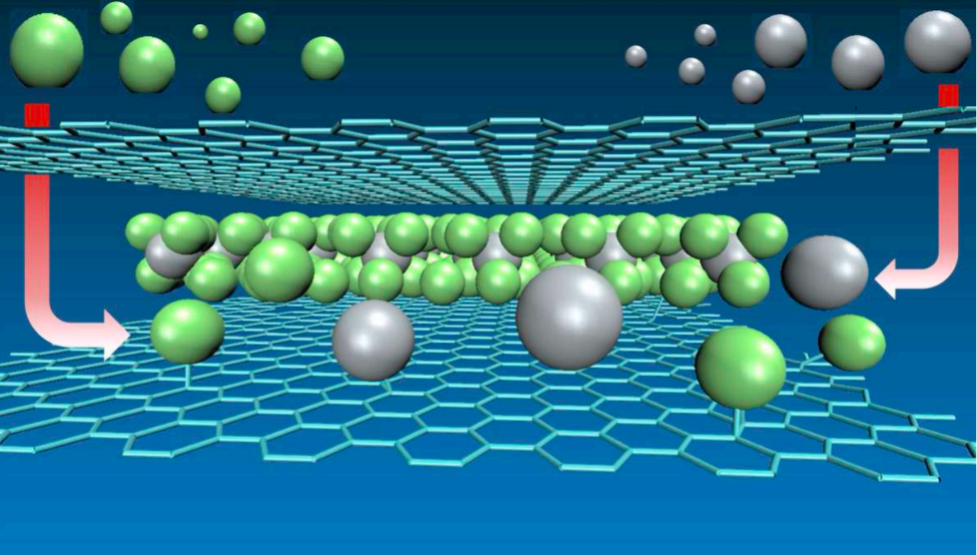

Bilayer graphene (BLG) has recently been used as a tool to stabilize encapsulated single sheets of various layered materials and tune their properties. It was also discovered that the protecting action of graphene sheets makes it possible to synthesize completely new two-dimensional materials (2DMs) inside the BLG by intercalating various atoms and molecules. In comparison to the bulk graphite, BLG allows for easier intercalation and a much larger increase in the interlayer separation of the sheets. Moreover, it enables studying the atomic structure of the intercalated 2DM by using high-resolution transmission electron microscopy. In this review, we summarize the recent progress in this area, with a special focus on new materials created inside BLG. We compare the experimental findings with the theoretical predictions, pay special attention to the discrepancies, and outline the challenges in the field. Finally, we discuss unique opportunities offered by intercalation into 2DMs beyond graphene and their heterostructures.

Intercalation of various atom and molecular species into layered materials has recently been at the forefront of research in materials science as an important phenomenon directly relevant to energy/ion storage^1,2^ and tuning the electronic properties of the materials.^3,4^ In particular, intercalation and deintercalation of alkali metal (AM) atoms into graphitic carbon systems,^5^ inorganic layered materials^2^ and their heterostructures^6^ has been extensively studied in the context of anode operation in electric batteries. Intercalation of AM atoms into transition metal dichalcogenides (TMDs) can be also used to induce phase transformations in these systems.^7^ The intercalation of organic and inorganic molecules into TMDs and other van der Waals (vdW) materials has been demonstrated to be a powerful tool for tuning material properties, e.g., adding superconductivity and magnetism, see refs (3 and 4) for an overview.

The isolation of graphene sheets and other two-dimensional materials (2DMs) followed by the development of vdW heterostructures opens new directions in this research. The chemical inertness and mechanical robustness of graphene allowed one to use graphene as a substrate to grow and stabilize essentially free-standing 2DMs. Moreover, in addition to 2DMs, which have layered bulk counterparts, such as TMDs,^8,9^ and can be exfoliated by various techniques, insulating 2D silica,^10^ semiconducting PdI_2_,^11^ or metallic AuCu,^12^ which are either not layered in the bulk form^10,12^ or are difficult to exfoliate,^11^ were manufactured. Moreover, as graphene is stable under electron beam at electron energies below 80 keV,^13^ the direct information on the atomic structure can be obtained using high-resolution transmission electron microscopy (HR-TEM) due to the graphene’s “transparency” to the electrons.^14^

2DMs,^15−19^ liquids,^20^ and soft materials like DNA strands^21^ on graphene can further be stabilized by placing another graphene sheet on top. It was also discovered that the protecting action of graphene can not only prevent marginally stable 2D materials from decomposition in air, but together with the electron beam facilitate phase transformations in the materials thus obtaining completely new 2D systems.^22−24^ Moreover, direct intercalation of atomic or molecular species^22,23,25^ or ion implantation^26^ into bilayer graphene (BLG) can be done. This can give rise to formation of new phases, as apparently more material can be intercalated into BLG than between graphene sheets in graphite, as it is much easier to “push” two free-standing layers away than those in the bulk layered counterpart. At the same time, pressure up to one GPa can be exerted on the encapsulated 2DMs,^27^ especially close to the material edges in graphene pockets, which, together with charge transfer to/from graphene, can affect material structure and properties.

The effects of confinement and also higher flexibility (that is, the ability to provide more space) of bilayer graphene as compared to graphite gave rise to unexpected behavior of intercalants. For example, formation of multilayer AM structures has been observed in BLG,^28,29^ contrary to a widespread belief that alkali metal atoms intercalated into layered materials form single-layer structures only. Exotic 2DMs can also be created by intercalation of atoms under graphene on metal substrates^30^ or under graphene on a monocrystalline SiC surface,^31−33^ but the materials are normally not free-standing, but covalently bonded to the substrate in this case, and besides direct TEM characterization of the material is not possible. On the contrary, after intercalation into BLG on a TEM grid, the atomic structure of the system can be thoroughly investigated. Many other materials can be potentially grown and characterized by using this approach.

In this mini-review, we summarize the progress in the material development inside BLG and outline the challenges both experiment and theory are facing at the moment. Our ultimate goal is to attract the attention of the scientific community to this relatively simple but efficient approach to make new 2DMs inside BLG and potentially other bilayers by utilizing the stabilization effects of the protective layers and also tuning their properties by charge transfer, pressure buildup related to spatial confinement, and electron beam irradiation. We do not focus on the synthesis details, as the methods are very close to the standard techniques used for the intercalation of various species into layered materials, see refs (3, 4, and 34).

We start by briefly outlining the approaches that can be employed to create 2D materials confined inside BLG, and then we dwell upon the materials that have been manufactured. Finally, we discuss unique opportunities offered by the intercalation into 2DMs beyond graphene and their heterostructures.

Several approaches can be used to develop new materials or unusual spatially confined phases of known materials in BLG. Figure 1 schematically presents the approaches. The atomic or molecular species can directly be intercalated into BLG at room or elevated temperatures, Figure 1(a), followed by chemical reactions in a confined space, which can also be stimulated by the electron beam.

**Figure 1:**
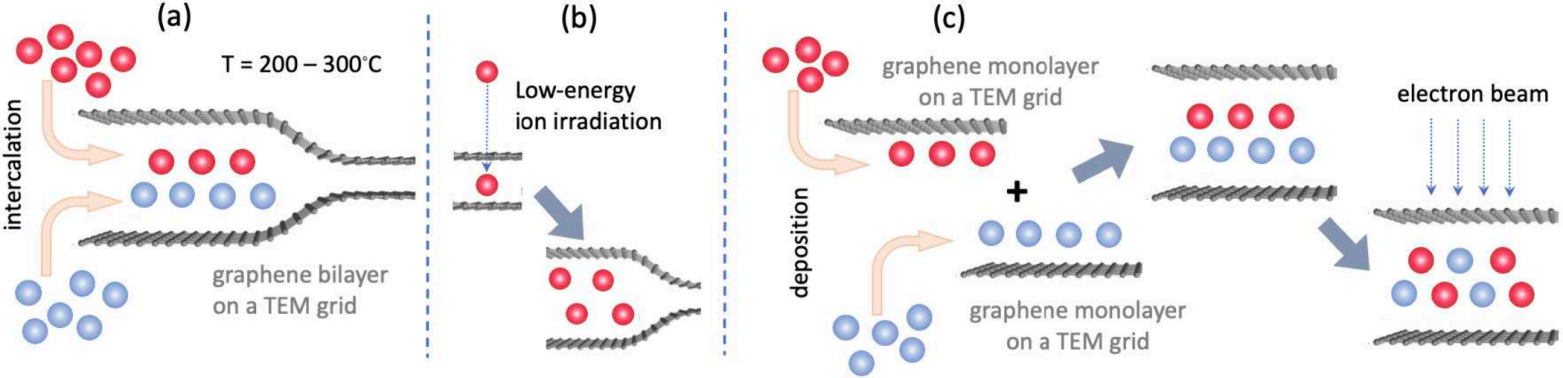
Schematic representation of the approaches that can be used to manufacture new 2D materials or unusual spatially confined phases of known materials in BLG on a TEM grid. (a) Intercalation of atoms and molecules into BLG at elevated temperatures. (b) Direct low-energy ion implantation into BLG possibly combined with mild annealing. (c) Deposition of materials on single-layer graphene on TEM grids, followed by making a “sandwich.” The material can be further transformed due to exposure to the electron beam in a TEM, as in the case of direct intercalation.

Low-energy ion implantation into BLG can also be employed, as shown in Figure 1(b). The choice of the ion energy is obviously of utmost importance, as ions would go through the system when the energy is too high, and would bounce back when the energy is too low.

Another approach is the deposition of materials on single-layer graphene on TEM grids, followed by making a ’sandwich’, as illustrated in Figure 1(c). The structure of the material can be further transformed due to exposure to the electron beam in a TEM, as in the case of direct intercalation.

Finally, AM atoms can be driven into the BLG by applying voltage to the systems. The setup involves BLG either on a substrate or a TEM grid, electrolyte, and AM ions, as discussed below.

Having introduced the methods that can be used to manufacture materials inside BLG, we move on to the specific materials produced using the approaches discussed above. The results obtained so far are also summarized in Table 1.

**Table 1:** Summary of the Encapsulated 2D Materials with Unusual Structures Obtained so Far

2D material manufactured inside BLG	Synthesis method	Characterization techniques	Electron-beam induced transformations	Reference in the paper
CdI	Wet chemical process involving liquid phase filtration of graphene oxide combined with metal chloride solution and hydroiodic acid.	TEM/STEM/EELS, electron diffraction, X-ray absorption spectroscopy	No	
AgI				(35)
NiI_2_				
MoCl_*x*_	Vapor transport method of intercalation: Heating of MoCl_5_ and BLG in a vacuum-sealed glass tube.	TEM/STEM/EELS	Yes, formation of FeCl_*x*_ phases with different stoichiometries and morphologies	(23)
FeCl_*x*_	Vapor transport method of intercalation: Heating of anhydrous FeCl_3_ and BLG in a vacuum sealed borosilicate ampule.	TEM/STEM, Raman spectroscopy	Yes, formation of FeOCl	(25)
FeOCl				
AlCl_3_	Vapor transport method of intercalation: Heating of AlCl_3_, and CuCl_2_ powders afterMoCl_5_ and BLG in a vacuum-sealed glass tube.	STEM/EELS, Raman spectroscopy	Yes, formation of AlCl_3_ phases with different morphologies	(22)
CuCl_2_ and their heterostructures				
Multilayer Li structures	Li/electrolyte and BLG forming an electrochemical cell.	TEM/EELS, electrical transport measurements	No	(28)
K, Rb, and Cs bilayers	Vapor phase intercalation process	TEM/EELS, Raman spectroscopy, electrical transport measurements	No	(29)
2D Pd	Electrochemical growth of 2D Pd between graphene oxide layers	TEM/AFM	No	(48)
2D Ar clusters	Direct ion implantation into BLG	TEM/STEM/EELS	No	(26)

## Covalently Bonded Inorganic 2D Materials

**Figure 2:**
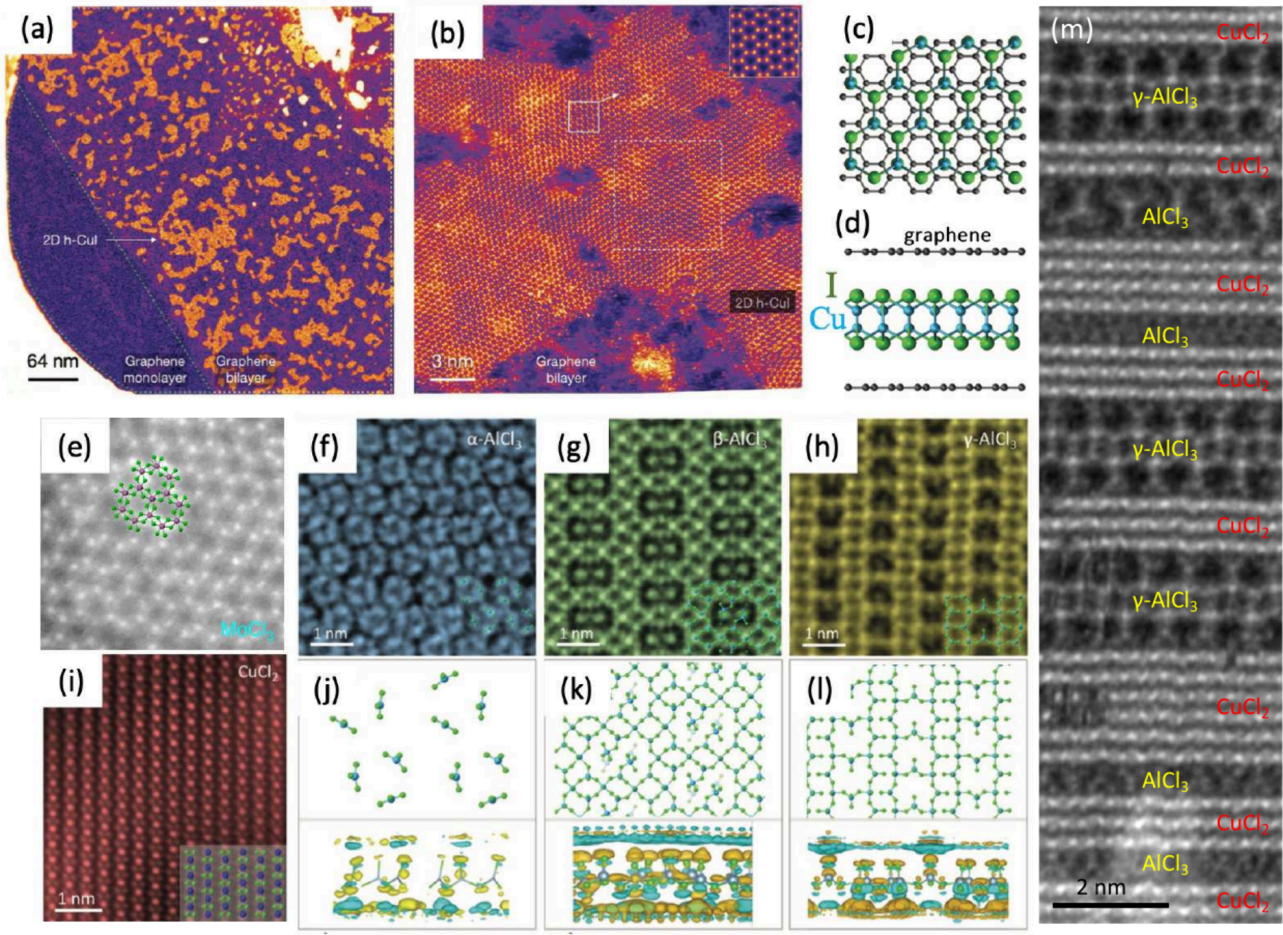
Inorganic 2D materials between the graphene sheets of BLG. (a) TEM image of monolayer h-CuI crystals encapsulated in BLG. Note that no h-CuI is visible on the monolayer area on the left-hand side. (b) Atomically resolved TEM image of a single 2D h-CuI crystal with a magnifying inset in the top right corner. (c,d) Top and side views of the atomic structure of h-CuI, as revealed by DFT calculations. Reproduced with permission from ref (35). (e–i) 2D MCl_*x*_ structures (M = Mo, Al, Cu) in BLG. (j–l) Atomic structure and charge transfer for AlCl_3_ phases obtained using first-principles calculations. (m) AlCl_3_/CuCl_2_ heterostructures. Panel (e) reproduced with permission from ref (23) and panels (f–m) from ref (22).

Single layers of CuI, a material that normally only occurs in the layered form at high temperatures between 645 and 675 K, were manufactured by a single-step wet chemical process involving liquid phase filtration of graphene oxide combined with a metal chloride solution and hydroiodic acid.^35^ Graphene oxide was eventually converted to BLG, and the materials encapsulated between graphene sheets were stable at ambient conditions. This new 2DM was named by the authors as hexagonal copper iodide (2D h-CuI). A TEM image of monolayer h-CuI crystals encapsulated in BLG is shown in Figure 2(a). Note that no h-CuI was observed in the monolayer graphene area, which emphasizes the importance of encapsulation. Atomically resolved TEM images of a single 2D h-CuI crystal with a magnifying inset in the top right corner are presented in Figure 2(b). The material was characterized by various techniques, including a combination of atomic-resolution scanning TEM (STEM) and ptychographic imaging, electron diffraction, X-ray absorption spectroscopy, and spatially resolved electron energy loss spectroscopy (EELS). Together with the results of density functional theory (DFT) calculations, Figure 2(c,d), the experimental data provided insights into the atomic structure of the encapsulated CuI system. Moreover, using the same approach 2D silver iodide (AgI) and nickel iodide (NiI_2_) crystals were manufactured,^35^ thus pointing out that other exotic encapsulated 2D materials can also be grown.

Various phases of 2D metal chlorides with different stoichiometries were produced in BLG^22,23,25^ by intercalation. Specifically, MoCl_*x*_ sheets were intercalated into BLG on TEM grids by placing the grid and MoCl_5_ powder into glass tube and heating it to 250 °C for 12 h in a furnace.^23^ During the heating, the MoCl_5_ powder turned into gas and intercalated into the area between the graphene layers. The prepared samples were opened in a glovebox and transferred to the TEM with minimal exposure to the atmosphere to prevent oxidation. TEM characterization revealed that the intercalated material represents MoCl_3_ networks, Figure 2(e), MoCl_2_ chains, and Mo_5_Cl_10_ rings. Exposure to the electron beam and possibly charge transfer from graphene gave rise to giant lattice distortions and frequent structural transformations, which have never been observed in metal chloride systems.

Iron chloride molecules were intercalated into BLG.^25^ Two distinct intercalated 2D systems were found by using TEM/STEM and Raman spectroscopy. They were identified as monolayer FeCl_3_ and FeCl_2_. These structures are magnetic, which may offer a way to study magnetism in a system with reduced dimensionality. It was also found that the electron beam can convert the FeCl_3_ monolayer into FeClO monolayers with a rectangular lattice.

Other unprecedented 2D metal chloride structures were produced in BLG through intercalation of metal and chlorine atoms via chemical vapor transport inside a vacuum-sealed glass tube.^22^ In particular, several spatially confined 2D phases of AlCl_3_ distinct from their typical bulk forms were observed by using HR-TEM, along with the transformations between the phases likely induced by the electron beam. Figure 2(f–h) shows the STEM images of the phases of 2D AlCl_3_, while Figure 2(j–l) presents the atomic structure and charge transfer for AlCl_3_ as obtained using first-principles calculations, which confirmed the metastability of the atomic structures derived from the experimental images. They also provided insights into the electronic properties of the phases: They were found to range from insulators to semimetals. 2D CuCl_2_ sheets (which in fact are quasi-one-dimensional chains parallel to each other) were also synthesized, Figure 2(i).

Additionally, completely new hybrid systems were produced by cointercalation of different metal chlorides. Specifically, in-plane AlCl_3_/CuCl_2_ heterostructures were manufactured, Figure 2(m). The existence of polymorphic phases and their appearance during exposure to the electron beam stress the important role electron irradiation plays. It also hints at unique possibilities for fabricating new types of 2D materials with diverse electronic properties confined between graphene sheets. It should be pointed out, however, that the driving force behind the transformations between phases remains unclear.

## 2D Metals

**Figure 3:**
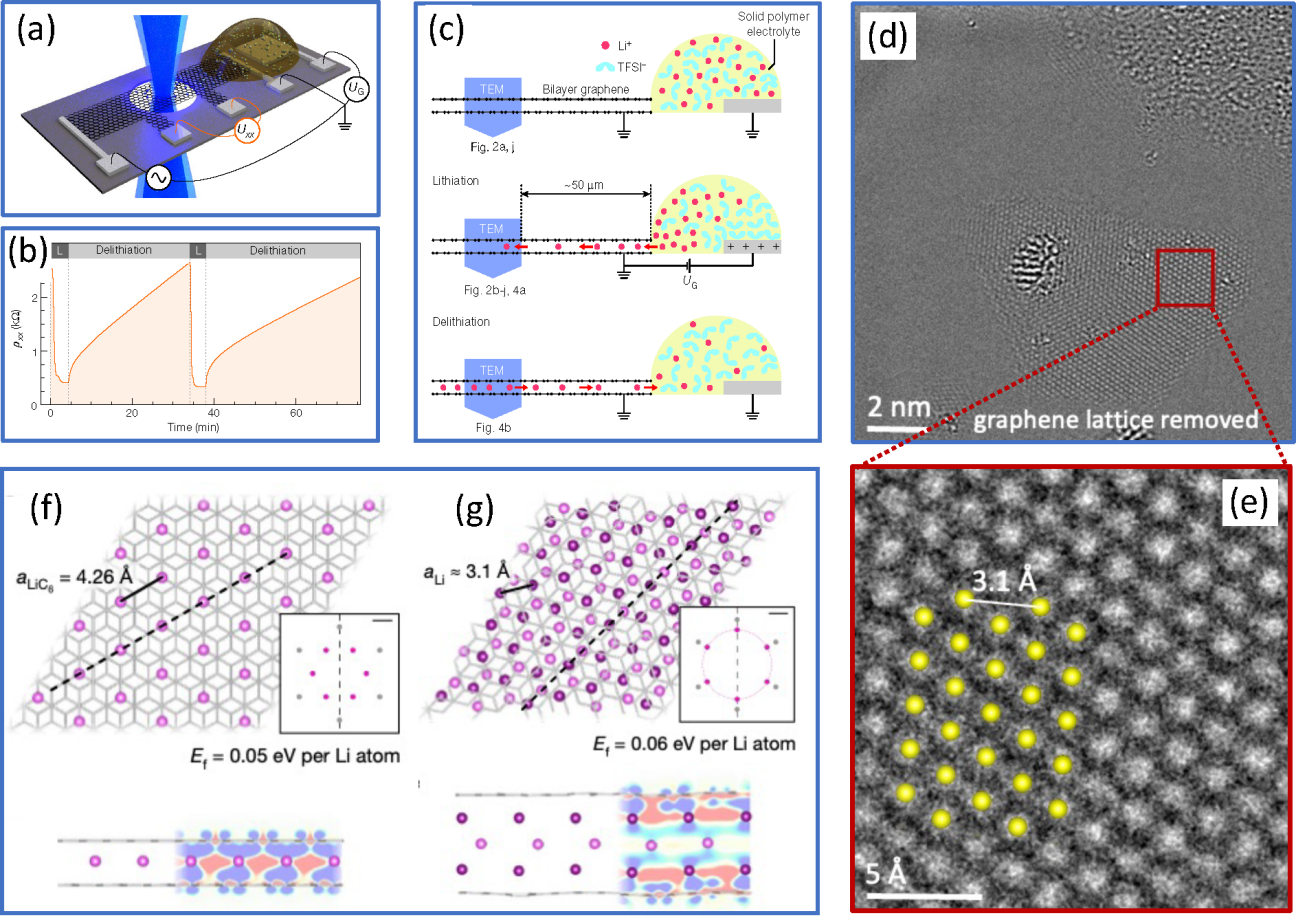
Li structures are in BLG. (a) Schematic of the device used in the *in situ* TEM experiments where multilayer Li structures were formed inside BLG.(b) Bilayer graphene resistivity measured *in situ* during two lithiation/delithiation cycles inside the TEM, schematically illustrated in panel (c). (d) Atomically resolved TEM image of a triangular Li crystal with graphene lattice removed. (e) Magnified view of the boxed area in panel (d). The positions of atoms are consistent with the multilayer Li fcc structure, but not with the √3 × √3*R*30° lattice. Panels (a–c, f) are reproduced with permission from ref (28), panels (d,e) from ref (41).

Intercalation of Li and other AM atoms into BLG has recently been studied both theoretically^5,36−38^ and experimentally.^5,28,29,39,40^ Specifically, a macroscopic three-dimensional BLG foam was recently manufactured,^39^ and its Li-storage capacity and intercalation kinetics were investigated. The results indicated that Li atoms can be stored only between the graphene sheets in BLG, not on the outer surfaces. At the same time, no microscopic data on the arrangement of Li atoms was obtained, but it was assumed, following other works^5,36−38^ on Li intercalation into BLG and graphite that Li atoms between graphene sheet form a √3 × √3*R*30° lattice, as illustrated in Figure 3(f).

Unexpected Li structures were observed in an *in situ* TEM study,^28^ which were claimed to be close-packed Li multilayers between graphene sheets. Figure 3(a) schematically shows the setup used in the experiment: BLG was deposited on a substrate with a hole at the center of the chip and contacted by several metallic electrodes, which made it possible to measure the resistivity of the device, which is different with and without intercalated Li atoms, Figure 3(b). The Li-ion (red spheres) conducting electrolyte (yellow) connected the BLG to a metallic counter electrode to form an electrochemical cell. The hole in the substrate allowed for *in situ* TEM observations of the lithiation and delithiation processes. Surprisingly, a dense network of Li atoms was found, Figure 3(e), which was not consistent with the √3 × √3*R*30° arrangement of atoms, Figure 3(f). The observations could be explained through the formation of close-packed multilayer structures, as shown in Figure 3(g). Follow-up studies^41^ indicated that Li in BLG mostly forms fcc lattice, consisting of up to ten layers. Simultaneous appearance of many triangular Li clusters was observed. An example is shown in Figure 3(d). This was explained through the nucleation of Li structures on vacancies in graphene, which have a higher probability to appear under the electron beam when Li atoms are present.^42^ The quasi-2D crystallites were found to be oriented in such a way that the (111) surface is facing graphene. This is surprising, as the (100) surface was shown to have the lowest formation energy in fcc Li crystals.^43^ However, the interface energy with graphene is the lowest for the (111) surface,^42^ which made such orientation energetically favorable.

The delithiation process was studied as well,^41^ and it was found that upon delithiation the impurity oxygen atoms initially embedded at octahedral interstitial positions inside the lithium crystals^42^ agglomerate at the edges of the crystals, thus giving rise to the formation of amorphous dendritic lithium oxide patches, where lithium ions are trapped. We note that unambiguous discrimination between the intercalated structures and those on the outer surface of BLG in the top view observation still remains a challenge, along with full characterization of the atomic structure of the compounds containing foreign atoms (oxygen, hydrogen, etc.). Further experiments involving EELS chemical analysis are required to differentiate between Li crystals and oxides or other compounds.

**Figure 4:**
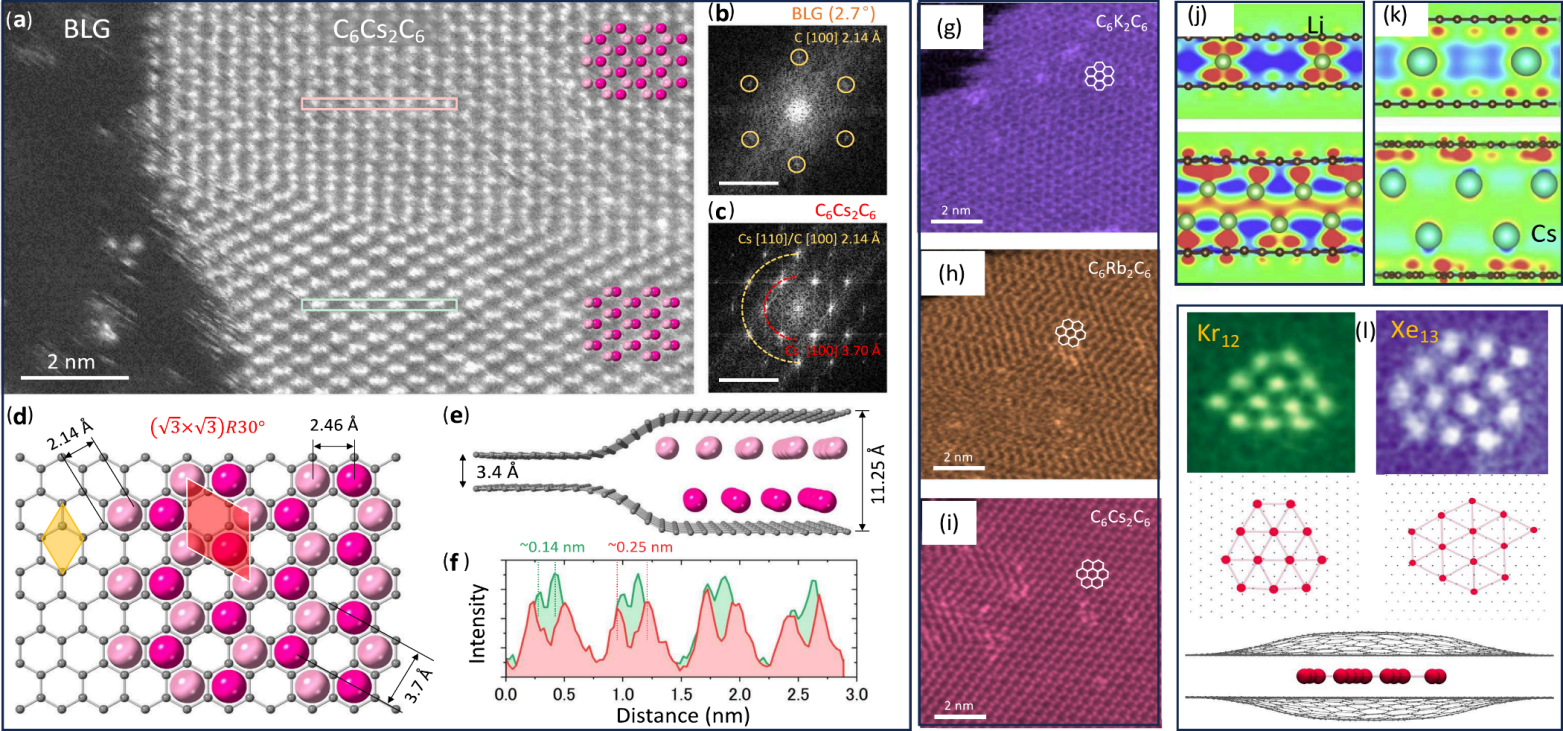
Unusual alkali metal and inert gas atom systems confined in the BLG. (a) STEM image of Cs-intercalated BLG displaying the C_6_Cs_2_C_6_ structure. (b,c) Fast Fourier transform image of BLG and Cs domains in panel (a), scale bar = 5 nm^–1^. (d) Top-view atomic model of C_6_Cs_2_C_6_. The yellow rhombus highlights a graphene (1 × 1) unit cell with *a* = *b* = 2.46 Å, while the red rhombus highlights the unit cell of the Cs lattice with √3 × √3*R*30° lattice, where *a* = *b* = 4.26 Å. (e) Side view of the C_6_Cs_2_C_6_ structure. (f) ADF profile of the hexagonal Cs layer along the pink and light green boxes in (a). The red profile displays the shortest distance between two Cs atoms as 0.25 nm, consistent with the atomic model shown in panel (d). The green profile shows the shorter distance between the Cs atoms, indicating the lateral displacement of the two Cs atomic plans. (g,i) TEM images of similar K, Rb, and Cs structures. Panels (a–i) reproduced with permission from ref (29). Charge difference (cross-section through Li (j) and Cs (k) atoms perpendicular to the BLG planes for single- and double-layer atoms structures. Note a buildup of the electron density between Li atoms and graphene, illustrating a substantial contribution to the bonding from covalent interaction. Red color corresponds to density build-up, blue to depletion. From ref (38). (l) Flat Kr and Xe clusters in BLG^26^ created by ion implantation.

The existence of multilayers of other AM atoms in BLG, MoS_2_ bilayers and their heterostructures was theoretically predicted.^38,44^ Although Li forms covalent bonds with graphitic carbon, Figure 4(j), while for other AMs the interaction is dominated by charge transfer^38,45,46^ between graphene and the AM atoms, multilayer structures for K are energetically favorable over single layers, and for Na, Rb, and Cs the energies are close.

Indeed double layers of K, Rb, and Cs inside BLG were observed^29^ later on. The intercalation of AM atoms into BLG on a TEM grid was done through a vapor phase intercalation process. TEM investigations revealed that the intercalated atoms form double layers inside BLG with the hcp stacking and have a C_6_M_2_C_6_ composition, Figure 4(a-i). A negative charge transferred from AM structure to graphene layers of approximately 1–1.5 × 10^14^*e*^–^cm^–2^ was determined by EELS, Raman, and electrical transport measurements, in agreement with the results of first-principles calculations, Figure 4(k). Studies on thicker graphene flakes showed that the double AM layers were absent in the graphite interior, primarily dominated by single-layer AM intercalation, which again emphasized the different behavior of intercalants in bilayers and bulk layered materials.

It should be pointed out, though, that the projected separations between the AM atoms were smaller than the DFT calculations predict.^38^ This can be explained by pressure exerted by graphene sheets in the direction parallel to the sheets, which can occur when the spacing between the sheets is only partially filled with AM atoms, as illustrated in Figure 4(e). This conjecture still remains to be confirmed by the calculations and/or further experiments.

Using the electrochemical deposition of Pd between graphene oxide sheets the growth of few-nm-thick Pd structures was achieved.^47^ The growth was self-limiting, which was a consequence of the strong interaction of Pd with the confining sheets, making the growth of bulk Pd energetically unfavorable. Liquid exfoliation of Pd sheets was then demonstrated along with their high efficiency in catalysis and electrocatalysis.

## Noble gas 2D structures

2D few-atom noble gas clusters were recently created^26^ between the layers in BLG using a very interesting approach: low-energy ion irradiation, as schematically illustrated in Figure 1(b). Specifically, Xe and Kr singly charged ions with ultralow energies of about 60 eV were implanted between suspended graphene sheets. Atomic-resolution characterization using STEM showed that graphene sheets remain mostly intact so that the implanted ions stay between graphene sheets and form clusters with geometries different from those of free-standing ones. The clusters were found to be flat due to the pressure of about 0.3 GPa coming from the graphene sheets. Their dynamics and atomic transformations at different temperatures were then investigated.

The successful creation of noble gas atom clusters by direct ion implantation indicates that this approach can possibly be used for the encapsulation of other atomic species. The strategies to “repair” graphene network should also be developed, by, e.g., adding hydrocarbon molecules. Moreover, as graphene is robust under electron beam (at electron energies below 80 keV^13^), exposure to the electron irradiation may stimulate chemical reactions and phase transformation in the confined area of BLG.

As evident from a brief summary of the results obtained so far, BLG is a unique platform to create novel 2DMs by intercalations, as it in comparison to graphite allows for easier intercalation and a much larger increase in the interlayer separation of the sheets. Potentially other 2DMs, such as h-BN or TMDs and their heterostructures, can be used. Another interesting direction is to create BLG with the controlled twist angle, which affects the behavior of the intercalated material.^48^ BLG, which is robust but transparent to the electron in the TEM, makes it possible to get direct microscopic information on the atomic structure of the encapsulated 2DMs. Moreover, new phases can appear due to electron irradiation, and the transformations can be followed *in situ*.

At the same time, many aspects of the intercalation, interaction of the encapsulated 2DMs with the protecting sheets, and effects of the electron beam still lack complete understanding. Specifically, the role of pressure and charge transfer should be fully clarified, along with the actual driving force for the observed phase transformations, which may be related to formation and dynamics of defects, as in the case of AlCl_3_.^22^ We note that the lateral sizes of the structures were rather small so that their characterization by larger scale methods was difficult, and one of the challenges is to increase the lateral size of the encapsulated structures. As for using ion implantation into BLG to create new materials, it is interesting to explore if this strategy can be extended from inert gas ions to other chemical elements, to find out what the optimum ion energies could be, and how inevitable damage to graphene sheets can be repaired.

Further developments in this field should enhance our understanding of the behavior of matter in confined space and suggest new ways for creating unprecedented 2D systems protected by graphene sheets or other robust 2DMs. As some structures, e.g., 2D iron chloride, are magnetic, this would allow studies on the magnetism in 2D systems, specifically in the context of information storage. Unique electronic properties and coexistence of different structures can allow for addressing a wide range of phenomena, such as Klein tunneling of Dirac-like Fermions or half-metal behavior. Controlled creation of defects in these systems, e.g., using electron beam, can open new avenues for the development of single-photon emitters, provided that insulating materials, e.g., h-BN are used for encapsulation. Overall, creation of 2D materials encapsulated into BLG should offer a unique opportunity to tune the properties of the system for specific applications such as photonics, quantum information, and energy storage.
